# School Well-Being and Drug Use in Adolescence

**DOI:** 10.3389/fpsyg.2020.01668

**Published:** 2020-07-31

**Authors:** Rosa Santibáñez, Josu Solabarrieta, Marta Ruiz-Narezo

**Affiliations:** ^1^Professor of Social Pedagogy and Diversity, Principal Researcher – INTERVENTION: Quality of Life and Social Inclusion, University of Deusto, Bilbao, Spain; ^2^Professor of Innovation and Management/Organizational Education, Member of Research Team Intervention: Quality of Life and Social Inclusion, Faculty of Psychology and Education, University of Deusto, Bilbao, Spain; ^3^University of Deusto, Bilbao, Spain; ^4^Associate Professor of Education, Member of Research Team Intervention: Quality of Life and Social Inclusion, University of Deusto, Bilbao, Spain

**Keywords:** school well-being, self-esteem, self-concept, gender, age, drug abuse, drug use

## Abstract

This research is part of the last study *Drugs and School IX* developed in the Basque Country (Spain) by the Instituto Deusto de Drogodependencias (Deusto Institute of Drug Addiction) of the University of Deusto (The study had the support of the Public Health and Addictions Directorate of the Deputy Health Ministry from the Health Department of the Basque Country.) and the data gathered by means of cluster sampling in two stages. The sample is made up of *N* = 6,007 girls and boys ranging from 12 to 22 years of age in secondary education, and the aims, on the basis of those parameters, are as follows: (1) describe the reality of drug consumption and some psychosocial variables in this sample, as well as analyze several relations between variables; (2) analyze the role of school well-being (SWB), self-esteem, and self-concept regarding consumption; (3) take a close look at the moderating role of age and gender on the relationship of school well-being, self-concept, and self-esteem with consumption; and (4) understand the existing interaction between all these variables, by studying the moderating role of self-esteem and self-concept in the influence of school well-being on consumption. With the use of a correlation, hierarchical regression, and mediation analysis with SPSS (v. 26) and Amos (v. 26) applications, three main conclusions were reached. Firstly, educational and academic well-being, academic self-concept, and self-esteem seem to play the role of protecting factors in adolescence, whereas assertiveness is linked to a higher consumption level. Secondly, academic self-concept has a mediating effect between well-being and consumption. Some of these relations are moderated by the variables of gender and age. Thirdly, age and gender are very relevant sociodemographic variables that must be taken into account in order to understand this phenomenon. Age has shown its covariant effect, which is especially relevant in the influence of academic well-being measured as being held back years. It has also proved to be important in order to understand its experiential or experimental and transitory character. Moreover, significant differences in consumptions have been found based on gender.

## Introduction

As their name suggests, risk conducts put at risk the well-being or the health of the person who engages in them or those in their surroundings. Adolescence is a complex period and, therefore, propitious for these kinds of conducts to arise (Skogen et al., 2019). However, despite the social alarm they generate, in most cases, their appearance during this period is linked to experimenting and, in the same way they appear, they disappear (Sánchez-Sosa et al., 2014; Batllori, 2016; Tena-Suck et al., 2018). Although the consumption of substances has decreased among the adolescent population in recent years in both Europe and Spain (ESPAD Group, 2016; Golpe et al., 2017) it is relevant to highlight some data. Alcohol, tobacco, and cannabis remain the most widely used substances and those most socially accepted. In Europe (ESPAD Group, 2016).

…, despite rather strict regulations on tobacco in most countries and on alcohol in some countries, adolescents still report relatively easy access to tobacco and alcohol. Moreover, trends over the past two decades indicate a closing of the gender gap in the use of tobacco and alcohol. The data suggest that cannabis remains an “established” drug. Although prevalence peaked in 2003 and decreased slightly thereafter, the prevalence rates in lifetime and current cannabis use are higher in 2015 than in 1995.

In Spain, alcohol is still the most consumed substance among the population between 14 and 18 years of age, according to the Observatorio Español de las Drogas y las Adicciones (2019). And, currently, the number of adolescents who smoke tobacco and cannabis is higher than the number of those who only smoke tobacco (Rial et al., 2019). The *Instituto Deusto de Drogodependencias* (Deusto Institute of Drug Addiction) has been researching this topic for over 30 years, as well as the variables that help understand this phenomenon. During the last academic year, it presented the last edition of the study *Drugs and School IX* (Instituto Deusto Drogodependencias, 2019). This series not only approaches this problem with a current and historical vision but also gives keys to guide the intervention of professionals linked to the educational environment. In this special issue on well-being and education, it would be very useful to get a better grasp on the connections between students’ school well-being (SWB) and substance abuse.

Experts define well-being as the subjective perception of satisfaction, happiness, a state where no negative conditions nor feelings exist (Keyes, 1998; Keyes et al., 2002). In the 1990s, Ryff and Keyes (1995) took a further step and tested their model of well-being that included different dimensions, such as autonomy, environmental mastery, personal growth, positive relations with others, purpose in life, and self-acceptance.

Some authors, such as Good and Willoughby (2014) have mentioned intrapersonal well-being (with oneself) and interpersonal well-being (with the outside world), but in any case, they always refer to subjective perceptions. This distinction can help us clarify the complex mosaic of studies and concepts used in this field. We have found studies on well-being in general, on school well-being, on educational well-being, on psychological well-being, on self-concept, and on self-esteem, all of them in relation to drug abuse. Educational agents are mainly interested in both dimensions of well-being: intrapersonal and interpersonal. These are the variables that are closest to the field of action of these agents.

Looking at intrapersonal well-being, the way we perceive ourselves from a cognitive point of view (self-concept) and the way we value ourselves from an emotional perspective (self-esteem) have been associated with a greater stability, behavioral and social coherence, and lesser risk and criminal, sexual, and consumption conducts (Collison et al., 2016; Kim et al., 2018). The studies on self-concept and self-esteem give us important keys to understanding well-being during adolescence. Ryff (1989) and Blanco and Díaz (2005) found a great correlation between self-acceptance (a subscale of well-being) and self-esteem (Rosenberg, 1965). Well-being seems to be broader and correlated with self-concept and self-esteem (Sarkova et al., 2014; Páramo et al., 2015). Although this logic may seem simple and linear, the relations are complex, and the results regarding substance consumption during adolescence are not conclusive (Fuentes et al., 2011; McKay et al., 2011, 2012). The reasons that may explain this are of varied nature. They may be related to the evolutionary period, which implies changes and possible incoherences that are typical at this stage. It may also be related to methodological matters and to the diversity of instruments used in research throughout the last decade. In this sense, the studies that assess self-esteem and self-concept as a multidimensional construct claim that their behavior during adolescence is differentiated. Thus, self-esteem and self-concept, both family and academic related, play a protective role, while social self-esteem, on the contrary, plays a risk role (Cava et al., 2008). In opposition to these studies, those who have measured self-esteem as a unitary construct have found that self-esteem plays, in any case, a mediating role (DuBois and Silverthorn, 2004; McKay et al., 2011; Álvarez-García et al., 2019). This means that the effect of certain variables on consumption may not be direct but go through self-esteem. Therefore, low self-esteem facilitates implication in criminal conducts or other risk conducts such as consumption and would highlight the importance of other variables, such as the family, academic, and social context. Thus, Christens and Peterson (2012) found that greater social support in the family, among peers, and also in the academic environment increases not only self-esteem but also the protective factors for violent conducts (Lázaro-Visa et al., 2019) and substance consumption (Malonda et al., 2019).

From an interpersonal point of view, there are no doubts regarding the importance of the school as a place for learning and acquiring content and skills, but we often forget the reach of the role of academic success in the adolescent’s personal life. McKay et al. (2012) found that the best predictor of alcohol consumption was low academic self-efficacy (according to Bandura’s definition)^1^. Thus, when formal education does not comply with these functions, there is an increase in the probability of adolescents being engaged in risk conducts, disconnecting from the school environment, and an increase in the probability of early school leaving, which entail negative psychosocial effects (Li and Lerner, 2011). Some authors have indicated that certain risk conducts, such as a regular consumption of drugs, entail academic difficulties (Bandura, 1997; González de Audikana, 2008, 2016; Wheeler, 2010; González de Audikana and Laespada, 2014). School is considered to be a context where the adolescent constructs well-being (Cash et al., 2014; Sarkova et al., 2014) not only in terms of their academic achievement but also in their relationships with other peers (Connolly et al., 2015; De Boer et al., 2016) and in their relationship with their teachers, the climate (Maxwell et al., 2017), the rules for coexistence, the attitudes, and values (Wang and Eccles, 2012; Wang and Fredricks, 2014; EMCDDA, 2015). Wang and Fredricks (2014) indicated that young people with a low commitment to school showed a higher tendency toward antisocial conducts (Álvarez-García et al., 2019), substance consumption, and early school leaving (González de Audikana, 2016).

Some authors such as Konu and her team (Konu et al., 2002, 2015) referred to school well-being as a concept. Ours is made up of three dimensions: academic welfare (failure or repetition), educational welfare (relationships with teachers and involvement), and interpersonal welfare (absence of conflict with peers).

Sociodemographic variables are also key to understanding both the well-being and the consumption phenomena. In well-being studies, Keyes et al. (2002) and Benner and Wang (2015) mentioned the importance of understanding well-being always framed in and conditioned by variables such as age, gender, and educational status. Regarding consumption, there are numerous researches that prove the existence of a positive relation between age and the frequency and intensity of substance consumption (Peñafiel, 2009; McKay et al., 2011; Motos et al., 2015; Hernández-Serrano et al., 2016; Riquelme et al., 2018). Nevertheless, this relation is curvilinear; that is, it increases with age, but when a maximum point is reached (usually between the ages of 18 and 24), it descends (Peñafiel, 2009). There is also proof of consumption differences based on gender. Both legal and illegal substances are associated repeatedly with boys (García del Castillo et al., 2004; Peralta et al., 2010; Fuller-Thomson et al., 2013; Riquelme et al., 2018). However, this is changing, and a tendency toward homogenization of consumption patterns is occurring, to the extent that the figures in alcohol and tobacco consumption have become even for both genders (Jiménez-Rodrigo, 2008; Khan et al., 2014; Instituto Deusto Drogodependencias, 2019).

This research aims to (1) describe the reality of drug consumption and some psychosocial variables in this sample, as well as study several relations between variables related to; (2) analyze the role of school well-being, self-esteem, and self-concept regarding consumption; (3) take a close look at the moderating role of age and gender on the relationship of school well-being, self-concept, and self-esteem with consumption; and (4) understand the existing interaction between all these variables, by studying the mediating role of self-esteem and self-concept in the influence of school well-being on consumption.

## Materials and Methods

### Procedure and Participants

The *Instituto Deusto de Drogodependencias* of the Faculty of Psychology and Education (Universidad de Deusto) conducted primarily a survey on secondary school students (between 12 and 22 years of age) in the academic year 2016/2017 in the Basque Autonomous Community (Instituto Deusto Drogodependencias, 2019) as a continuation of the series started in academic year 1981/1982. The aim was to recognize the situation of drug consumption and a set of factors associated with consumption and to learn its historical evolution. The study had the support of the Public Health and Addictions Directorate of the Deputy Health Ministry from the Health Department of the Basque Country. The data gathered from that survey provided an adequate measurement of the variables in the research questions. Statistical analysis computer applications SPSS (v. 26) and Amos (v. 26) were used.

Descriptive statistics (percentages, mean, standard deviation, and skewness) and bivariate statistics (Pearson’s correlation coefficient, *t*-test, chi-squared, odds ratio, and ANOVA) were used for the analysis of the relation of sociodemographic variables with well-being at school, self-esteem, and self-concept and consumption, including *p*-value and effect size estimations (Cohen’s *d*, Cramer’s *V*, and eta-squared). Pearson’s correlation coefficients, partial correlations, and a hierarchical regression analysis were included for analyzing the relation between self-esteem and self-concept, well-being at school, and consumption. Splitting data in groups according to age and gender and calculating separate Pearson’s correlation coefficients (Aiken and West, 1991) allowed to describe effect moderation by age and gender. The mediating roles of self-concept and self-esteem required a specific path analysis and the calculation of direct and indirect standardized regression coefficients (beta). Goodness of fit in construct validity analysis and path analysis was tested using Tucker–Lewis index (TLI), comparative fit index (CFI), and the root mean square error of approximation (RMSEA).

A cluster sampling in two stages (a random selection of schools, followed by a random selection of classrooms) was carried out among the population enrolled in the secondary education in the Autonomous Community of the Basque Country. The sample is composed of 6,007 subjects; 45.4% are women, and their average age is 15.37 with a standard deviation of 2.18 years. The age range is between 12 and 22 years, and the age groups are represented in a balanced manner between 12 and 19; 59.8% study *Educación Secundaria Obligatoria* (compulsory secondary education; ESO), 21.1% *Bachillerato* (the last 2 years of secondary education), 13.1% study *Formación Profesional Media* (intermediate level vocational training; FPM), and 6% study *Formación Profesional Básica* (basic vocational training; FPB). Compared with the Spanish average educational indicators, the Autonomous Community of the Basque Country has a larger proportion of charter schools (48.4 vs. 25.7%), a higher budget for each student (9,054 vs. 5,607 euros), and lower repetition (5.5 vs. 8.7%) and early school leaving rates (7.0 vs. 18.3%) in secondary education (CEE, 2018; MEFP, 2019) but does not stand out in the average PISA scores (science 483 vs. 493, reading 491 vs. 496, and mathematics 492 vs. 486) (ISEI-IVEI, 2017).

The average score in the socioeconomic level variable of the families was 14.19, with a standard deviation of 4.70, and a skewness of −0.235; 16.4% are second-generation immigrants, and 1.4% are first-generation immigrants.

Of the sample, 42.4% study in the public system, and 57.5% in the private system; 56% study model D (mainly in Basque), 29.4% model A (mainly in Spanish), and 14.6% model B (intermediate level, which combines Basque and Spanish); 18.4% have been held back a year, and 10.3% have been held back two or more years. Most of the people from the sample (69.8%) did not fail any subjects in the month of June of the previous academic year, whereas 12.1% failed one subject, 10.5% two subjects, and 7.6% three or more subjects. When they were asked how much money they had for their personal expenses, 65.9% indicated they had up to 10 euros per week; 21.3%, between 11 and 20 euros; 10.2%, between 21 and 50 euros; and 2.6%, 51 euros or more.

Regarding consumption levels, 32.3% indicated that they do not consume any substances; 33% were classified in the category of alcohol consumption, 26.3% in the category of cannabis, 5.1% in stimulant substances (cocaine, amphetamine or speed, and ecstasy or similar substances), and 3.3% in minority substances (heroin and LSD or similar substances). It must be taken into account that these levels frequently contain cumulative consumptions (e.g., a big part of those who consume cannabis also consume alcohol).

### Measurements

The sociodemographic data collected were age, gender, social level (combining the type of work and studies of the parents), the amount of money available for their personal expenses, and the educational cycle taken (*Educación Secundaria Obligatoria*, *Bachillerato*, FPB, and *Formación Profesional Media*).

Three dimensions of school well-being were considered. Educational well-being indicates the perception of the quality of students’ relationship with the teachers and their involvement in the learning process (e.g., “I enjoy carrying out my duties as a student” or “If I have any problems I know that I can go see a teacher”). It has a 6-point Likert scale: 1 = “Strongly disagree,” 2 = “Disagree,” 3 = “Slightly disagree,” 4 = “Slightly agree,” 5 = “Agree,” and 6 = “Strongly agree.” The value of Cronbach’s alpha coefficient was 0.680, but a confirmatory factor analysis (CFA) produced acceptable indices (TLI = 0.910; CFI = 0.976; RMSEA = 0.065). Academic well-being comprises the degree of adjustment to the formal academic requirements, indicated by grade repetition and the number of failed subjects. These questions were direct, asking for the number of years they had been held back, with three possible responses (1 = “No, I have not been held back,” 2 = “I have been held back a year”, and 3 = “I have been held back two or more years”), and the number of subjects they had failed in the final assessment of the previous year, with four possible responses (1 = “I passed everything,” 2 = “I failed one subject,” 3 = “I failed 2 or 3 subjects,” or 4 = “I failed 4 or more subjects”). Interpersonal well-being at the school corresponds to their perception of the quality of their relationship with their classmates and the absence of conflicts. It was measured by three items from the *Escala Multidimensional Breve de Ajuste Escolar* (Moral et al., 2010) (e.g., “I have problems with my classmates”) using a 6-point Likert scale. In this case, the value of Cronbach’s alpha coefficient for internal consistency was 0.750.

Three variables regarding self-esteem and self-concept were also collected. The Rosenberg (1965) Self-esteem scale was used, composed of 10 items (e.g., “On the whole, I am satisfied with myself.”) with a 4-point Likert scale (1 = “Strongly agree,” 2 = “Agree,” 3 = “Disagree,” and 4 = “Strongly disagree”). In this sample, Cronbach’s alpha was 0.768. Construct validity was analyzed by testing diverse models through CFA, given the complexity of this measurement. In general, this scale does not provide a simple one-dimensional measurement but tends to show differences when the question is phrased in an affirmative or negative sense. Some researches point at the possibility of each type of questions measuring different constructs, where the questions posed in a negative sense would reveal a construct related to depressive symptoms and self-deprecation. However, other researches indicate that the two-factor model is an artifact that is the result of the phrasing of questions. Greenberger et al. (2003) observed that when the questions are rephrased in the same direction, a one-dimensional scale is obtained. Huang and Dong (2012) recommend a solution with a single dimension, based on the revision of 23 studies and 80 samples.

This effect may be reflected in the measurement models in two ways: by adding covariance parameters between the questions asked in a specific direction or by including a method factor (effect of a positive or a negative formulation of the questions). This last technique is backed by a multi-risk multi-method approach, accepting the existence of a latent variable, which corresponds to the effect of the phrasing of the question in a specific direction. If the theoretical irrelevance of this variable in the model proposed is accepted with certainty, it is possible to acknowledge it in the measurement model but to ignore it afterward in the explanatory models (Little et al., 2002; Chen et al., 2007). In the present study, several models have been contrasted to obtain goodness-of-fit indexes that appear on Table 1, and the third model had the best indexes.

**TABLE 1:** Self-esteem scale CFA models’ goodness-of-fit indexes.

	CFI	TLI	RMSEA	90% CI
1. One factor model	0.745	0.599	0.119	(0.116; 0.123)
2. One factor model, with covariance parameters between negatively worded items	0.942	0.873	0.067	(0.063; 0.071)
3. One factor model, with covariance parameters between positively worded items	0.978	0.952	0.041	(0.037; 0.045)
4. One factor, adding a Methodology variable (wording negatively factor)	0.941	0.892	0.062	(0.058; 0.066)

*CFA, confirmatory factor analysis; TLI, Tucker–Lewis index; CFI, comparative fit index; RMSEA, root mean square error of approximation.*

Academic self-concept was measured by two items from the *Escala Multidimensional Breve de Ajuste Escolar* (School Adjustment Short Multidimensional Scale) (Moral et al., 2010) (e.g., “I think that I am a good student”) with a 6-point Likert scale (alpha = 0.819). Social self-concept was measured by six items from the *Self-Perception Profile for Adolescents* (*Social competence and Close friendship subscales*) by Harter (2012) on their perception of their capacity to make friends (e.g., “I find it easy to make friends”), in a 9-point semantic differential scale (e.g., 1 = “I find it easy to make friends” to 9 = “I find it hard to make friends”); Cronbach’s alpha obtained was 0.805, and CFA results were adequate (TLI = 0.962; CFI = 0.0987; RMSEA = 0.058).

Assertiveness was measured with a reduced version of *Rathus Assertiveness Schedule* (Rathus, 1973) composed of seven items (e.g., “If a relative were annoying me, I would tell them, even if this makes them angry”) with a 9-point semantic differential scale (e.g., 1 = “If a close and respected relative were annoying me, I would tell them, even if this makes them angry” to 9 = “If a close and respected relative were annoying me, I would smother my feelings rather than express my annoyance”). Cronbach’s alpha value was 0.810, and CFA results were good (TLI = 0.947; CFI = 0.975; RMSEA = 0.045).

**FIGURE 1:**
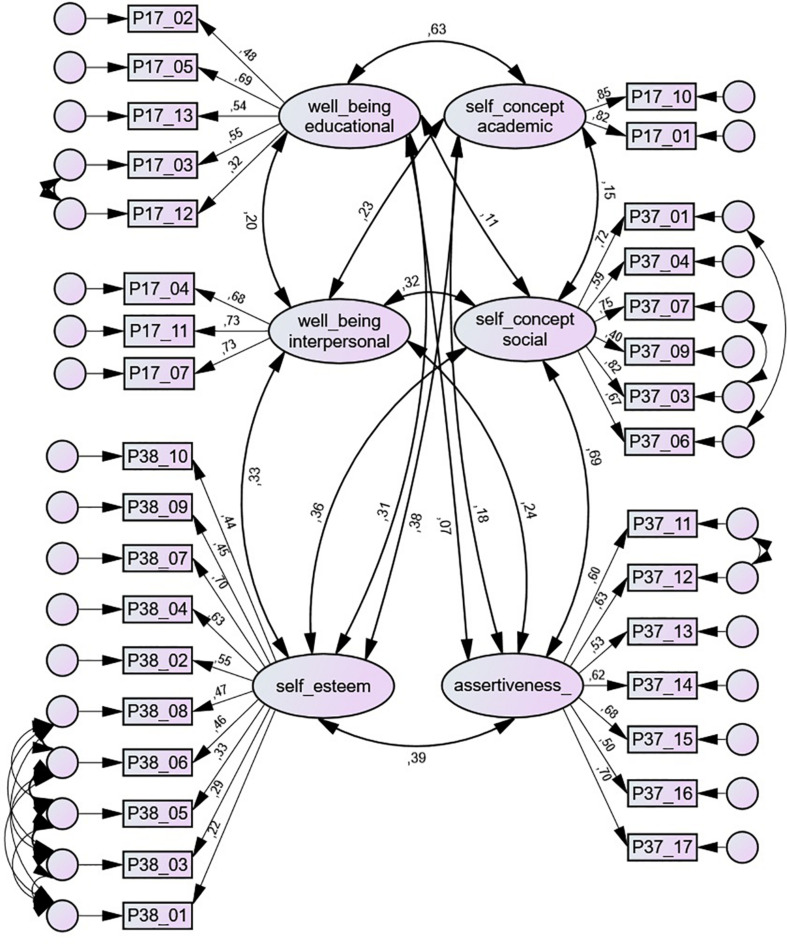
Measurement model with scales used.

The whole measurement model of scale scores (Figure 1) produced adequate fit indexes (TLI = 0.915; CFI = 0.929; RMSEA = 0.037; standardized root mean square residual (SRMR) = 0.0498).

Substance consumption was measured by asking the question “On how many occasions, if any, have you consumed during the past month?” The question was repeated and adapted to each substance, differentiating the consumption of alcohol (referring to being drunk, “On how many occasions, if any, have you been drunk from the consumption of alcoholic drinks in the past month?”), cannabis, stimulant substances (cocaine, amphetamine or speed, and ecstasy or similar substances), and minority substances (heroin and LSD or similar substances). The questionnaire offered seven possible responses (0 = “Never,” 1 = “1–2 times,” 2 = “3–5 times,” 3 = “6–9 times,” 4 = “10–19 times,” 5 = “20–39 times,” and 6 = “40 or more times”) for each substance. In some analyses, the variable was recoded into dichotomous (0 = “Never” and 1 = “At least on one occasion”). Afterward, several markings were used to calculate a single global indicator with five consumption levels, using a scale based on a study by González de Audikana (2017) which differentiates five drug consumption levels: no consumption, conventional substance consumption (alcohol), cannabis consumption, stimulant substance consumption (cocaine, amphetamines or speed, and ecstasy or similar substances), and minority substance consumption (heroin and LSD or similar substances).

## Results

The research intends to analyze the following:

1. The relation of some sociodemographic variables (gender, age, and stage) and well-being at school, self-esteem, and self-concept and consumption.
2. The relation between self-esteem and self-concept, well-being at school, and consumption.
3. The moderating roles of age and gender on the relation between consumption and well-being at school, self-esteem, and self-concept.
4. The mediating roles of self-esteem and self-concept in the influence of school well-being on consumption.

First of all, the relation of some sociodemographic variables (gender, age, and educational stage) and well-being at school, self-esteem, and self-concept and consumption was analyzed. As may be observed in Table 2, the level of educational well-being is higher among girls (*t* = −7.404; *p* = 0.000; Cohen’s *d* = −0.19), in *Formación Profesional Media* (FPM) and in FPB, and there is a U-shaped tendency throughout the different stages. Academic well-being, measured using indicators such as the number of failing grades and the number of years having been held back, is more unfavorable among boys, in FPB and FPM, and naturally shows a cumulative effect throughout the years.

**TABLE 2:** School well-being, self-concept, self-esteem, and drug use means and percentages by gender, educational stage, and age.

		School well-being Educational	School well-being Academic—fails	School well-being Academic—repet.	Self-concept Academic	Self-esteem	Self-concept Social	Assertiveness	0 No drug use (%)	1 Alcohol (%)	2 Cannabis (%)	3 Stimulant (%)	4 Minority (%)	1 Alcohol (%)	2 Cannabis (%)	3 Stimulant (%)	4 Minority (%)
Gender	0 male	3.93	0.67	0.48	4.20	3.09	6.77	7.30	31	30	28	6	5	22.5	21.7	4.4	1.2
	1 female	4.12	0.43	0.28	4.61	3.03	6.52	7.09	34	37	24	3	2	19.1	13.0	1.6	0.5
Educational stage	1 ESO	3.98	0.54	0.23	4.46	3.06	6.72	7.21	48	33	15	2	2	10.4	8.9	1.4	0.7
	2 BACHILLER	3.85	0.37	0.16	4.35	3.06	6.57	7.21	8	42	44	3	2	37.7	25.1	3.2	0.9
	3 FPBASICA	4.12	1.31	1.36	4.05	2.97	6.53	7.08	16	20	37	16	10	32.2	38.0	8.3	2.5
	4 FPMEDIA	4.41	0.61	1.03	4.25	3.07	6.57	7.23	8	23	43	16	10	38.0	37.4	9.1	0.9
Age	12	4.46	0.24	0.02	4.93	3.12	6.99	7.34	86	11	2	1	1	1.4	0.7	1.1	0.9
	13	4.10	0.42	0.10	4.69	3.08	6.78	7.20	67	26	5	1	1	4.0	2.7	0.7	0.9
	14	3.79	0.49	0.14	4.40	3.06	6.65	7.17	42	41	14	2	1	9.5	7.7	1.0	0.2
	15	3.86	0.64	0.26	4.29	3.03	6.64	7.18	27	42	26	3	2	16.1	14.5	1.3	0.4
	16	3.88	0.73	0.38	4.20	3.03	6.52	7.18	14	40	39	3	4	30.2	24.7	2.8	1.5
	17	3.94	0.64	0.56	4.24	3.06	6.58	7.21	10	35	43	8	4	36.1	29.4	4.9	0.9
	18	4.14	0.69	0.97	4.16	3.04	6.71	7.20	8	30	44	11	8	40.0	34.7	6.2	1.1
	19	4.36	0.65	1.24	4.17	3.06	6.52	7.33	8	20	41	21	9	39.9	42.7	11.3	1.9
	20	4.51	0.40	1.16	4.28	3.08	6.39	7.15	9	15	38	22	16	44.7	40.4	16.7	2.6
	21	4.56	0.37	1.24	4.42	3.12	6.67	7.21	9	16	52	14	10	39.0	20.3	5.1	0.0
	22	4.43	0.48	1.24	4.49	3.06	6.41	6.90	12	26	26	19	16	30.2	30.2	12.7	1.6

*ESO, Educación Secundaria Obligatoria; FPB, Formación Profesional Básica; FPM, Formación Profesional Media.*

Academic self-concept is higher among girls (*t* = −13.158; *p* = 0.000; *d* = 0.34) in *Bachillerato*, as opposed to FPB, and a decreasing tendency is observed with age. The average self-esteem is slightly higher among boys (*t* = 4.527; *p* = 0.000; *d* = 0.12), slightly lower in FPB, and remains stable throughout the years. Social self-concept is higher among boys (*t* = 6.892; *p* = 0.000; *d* = 0.18), descends after ESO, and has a certain decreasing tendency with age. Assertiveness is higher among boys, lower in FPB, and shows stability at different ages.

Regarding the highest drug use level (alcohol, cannabis, stimulant substances, and minority substances) reached by each person and gender (*χ*^2^ = 96.938; *p* = 0.000; Cramer’s *V* = 0.131), there are more girls in the no-consumption group as well as in the alcohol-consumption group. On the other hand, there is a higher percentage of boys who consume cannabis and other substances.

When the consumption prevalence of each substance in the last month is analyzed, the proportion of boys having gotten drunk (22.5%) is only slightly larger than that of girls (19.1%) (OR = 0.811; 95% CI 0.715–0.921). However, for the remaining substances, the proportion of boys who have consumed them is much larger (21.7% vs. 13.0% for cannabis, OR = 0.540, 95% CI 0.469–0.621; 4.4% vs. 1.6% for stimulant substances, OR = 0.348, 95% CI 0.246–0.493; 1.2% vs. 0.5% for minority substances, OR = 0.440, 95% CI 0.238–0.814).

When comparing the educational stages, *Bachillerato* and FPM, which correspond to the groups with older students, show the greatest prevalence of monthly consumption of alcohol. The consumption indicated in FPB is remarkable. The proportion of cannabis and stimulant-substance consumers is larger in FPB and FPM. Minority substances are especially present in FPB.

**FIGURE 2:**
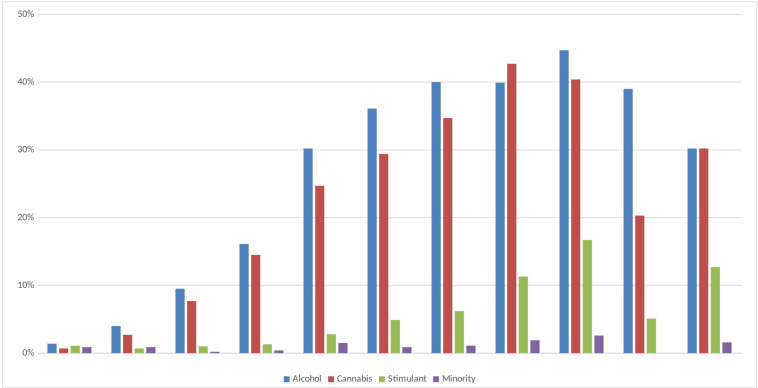
Drug use distribution by age (having used drugs in the last month).

The drug use prevalence indicators are higher the older the age, with the highest prevalence observed around age 19 or 20, and with lower prevalence at the following ages (Figure 2). The typical ages for the highest irruption of each substance are different: younger in alcohol consumption, followed by cannabis, stimulant substances, and minority substances.

**FIGURE 3:**
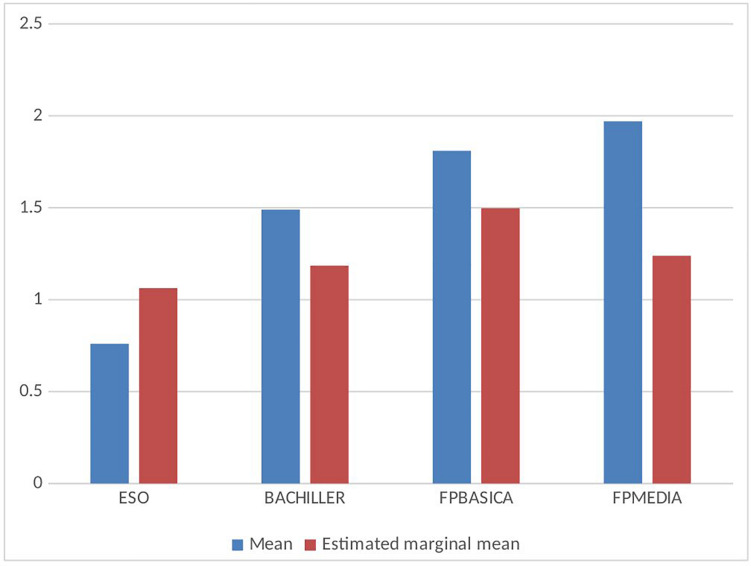
Mean and estimated marginal mean of drug use level at each educational stage.

The strong relation between consumption levels and age makes it advisable to control the effect of age when analyzing the relation of other variables with drug consumption. An example is the comparison of the average consumptions at the different school stages. Consumption is higher in FPM, FPB, *Bachillerato*, and ESO, in this order (*F* = 524.285; *p* = 0.000; *η*^2^ = 0.215). When controlling the effect of age, in Figure 3, estimated marginal means are very different compared with observed means, and not so different from each other (*F* = 19.998; *p* = 0.000; *η*^2^ = 0.010). The confounding effect of age seems to have been hiding that the highest average is produced in FPB.

Secondly, the relation between well-being at school, self-esteem and self-concept, and consumption was analyzed. Table 3 shows the values of Pearson’s product-moment correlation coefficient between these variables. Zero-order correlations are represented under the diagonal, whereas partial correlations once the effect of age is controlled are shown over the diagonal. Great differences are observed between both types of correlation, which suggests that the shared effect of age underlies in a fair share of the apparent correlations between many variables.

**TABLE 3:** Zero-order correlations and partial correlations between main variables^3^.

		1–Age	2	3	4	5	6	7	8	9	10	11	12	13	14	15	16	17	18	19	20
2	Gender	–0.086		0.039	–0.117	0.101	–0.062	–0.055	0.092	–0.127	–0.126	0.029	0.180	–0.087	–0.109	–0.092	–0.071	–0.068	–0.148	–0.037	–0.006
3	Social level	–0.233	0.056		–0.032	0.116	–0.116	–0.079	–0.007	–0.178	–0.209	0.096	0.139	0.065	0.066	0.021	–0.006	0.020	–0.018	0.002	0.008
4	Money	0.449	–0.126	–0.137		–0.136	0.050	0.140	–0.009	0.093	0.177	–0.003	–0.086	0.038	0.087	0.063	0.121	0.134	0.197	0.139	–0.002
5	Bachillerato	0.334	0.055	0.038	0.069		–0.177	–0.595	–0.101	–0.152	–0.441	0.091	0.037	–0.007	–0.038	–0.009	–0.009	0.045	–0.080	–0.041	–0.020
6	FPB	0.164	–0.081	–0.147	0.112	–0.131		–0.175	0.025	0.153	0.318	–0.056	–0.035	0.002	–0.006	0.011	0.096	0.016	0.107	0.006	0.028
7	FPM	0.597	–0.079	–0.198	0.356	–0.200	–0.098		0.195	0.020	0.193	–0.039	0.016	0.009	0.020	0.012	0.005	0.000	0.078	0.036	–0.006
8	SWB—educational	0.012	0.096	–0.022	–0.017	–0.091	0.029	0.160		–0.153	–0.008	0.138	0.434	0.214	0.066	0.034	–0.241	–0.127	–0.177	–0.052	–0.040
9	SWB academic-fails	0.098	–0.125	–0.191	0.131	–0.102	0.197	0.020	–0.145		0.412	–0.114	–0.421	–0.119	–0.008	–0.019	0.138	0.084	0.149	0.032	0.027
10	SWB academic-repet.	0.488	–0.157	–0.288	0.336	–0.180	0.371	0.372	0.020	0.424		–0.135	–0.212	–0.022	0.000	0.031	0.108	0.050	0.187	0.085	0.023
11	SWB interpersonal	0.006	0.038	0.090	0.004	0.100	–0.099	–0.003	0.131	–0.148	–0.125		0.164	0.227	0.227	0.148	–0.008	0.002	–0.049	–0.029	–0.034
12	Self-concept academic	–0.149	0.168	0.167	–0.137	–0.014	–0.070	–0.044	0.424	–0.426	–0.256	0.176		0.293	0.088	0.121	–0.252	–0.126	–0.211	–0.047	–0.046
13	Self-esteem	–0.022	–0.060	0.064	0.021	0.005	–0.048	0.013	0.221	–0.137	–0.054	0.244	0.310		0.372	0.330	–0.075	–0.026	–0.059	0.012	0.015
14	Self-concept social	–0.072	–0.090	0.076	0.045	–0.031	–0.022	–0.022	0.060	–0.036	–0.031	0.235	0.115	0.353		0.522	0.077	0.076	0.052	0.020	0.013
15	Assertiveness	–0.014	–0.075	0.019	0.049	0.004	–0.022	0.008	0.032	–0.054	–0.003	0.185	0.133	0.347	0.559		0.074	0.055	0.039	0.026	–0.016
16	Drug use level	0.530	–0.108	–0.121	0.351	0.181	0.164	0.312	–0.200	0.175	0.346	0.007	–0.270	–0.078	0.026	0.053		0.311	0.466	0.266	0.192
17	Alcohol	0.293	–0.078	–0.045	0.257	0.127	0.086	0.179	–0.111	0.126	0.210	–0.027	–0.145	–0.031	0.042	0.033	0.411		0.337	0.194	0.092
18	Cannabis	0.300	–0.136	–0.080	0.318	0.016	0.181	0.235	–0.136	0.185	0.317	–0.039	–0.216	–0.037	0.033	0.044	0.541	0.385		0.244	0.070
19	Stimulant	0.114	–0.063	–0.021	0.143	0.012	0.066	0.079	–0.065	0.069	0.129	–0.063	–0.073	–0.023	–0.009	–0.004	0.311	0.234	0.283		0.189
20	Minority	0.006	–0.023	0.014	0.002	–0.009	0.032	–0.007	–0.048	0.041	0.041	–0.035	–0.042	–0.025	–0.018	–0.015	0.208	0.093	0.102	0.291	
	Mean	15.37	0.45	14.19	1.49	0.21	0.06	0.13	4.01	0.56	0.39	5.42	4.39	3.06	6.66	7.20	1.14	0.35	0.58	0.08	0.03
	Std. deviation	2.18	0.50	4.70	0.78	0.41	0.24	0.34	0.95	0.96	0.67	0.93	1.21	0.48	1.43	1.37	1.03	0.85	1.49	0.56	0.36

*FPB, Formación Profesional Básica; SWB, School Well-Being. Zero-order correlation coefficients under the diagonal and partial correlation (controlling for age) over the diagonal. These correlations are significant at the 0.05 level when they are >0.026 and at the 0.01 level when they are >0.035, for a two-tailed test.*

Educational well-being is positively correlated with academic self-concept (*r* = 0.424) and self-esteem (*r* = 0.221). Academic well-being, measured as the number of failed subjects, has a negative correlation with academic self-concept (*r* = −0.426) and with self-esteem (*r* = −0.137), but being held back years is less correlated with both variables. Interpersonal well-being at school is related to self-esteem (*r* = 0.244), social self-concept (*r* = 0.235), academic self-concept (*r* = 0.176), and assertiveness (*r* = 0.185). None of these correlations is significantly modified when controlling age.

Consumption levels are associated with academic well-being (being held back years *r* = 0.346, number of failed subjects *r* = 0.175), but this correlation changes substantially when age is controlled. Then the number of failed subjects is correlated with consumption (*r* = 0.138) more than with being held back years (*r* = 0.108). On another note, educational well-being shows partial correlation with consumption (*r* = −0.241) higher than the zero-order correlation (*r* = −0.200). All these correlations are stronger in the case of frequency of alcohol consumption, in comparison with the frequency of consumption of other substances. The level of consumption is not correlated with interpersonal well-being at school.

Academic self-concept predicts the consumption level (*r* = −0.270) much better than self-esteem (*r* = −0.078), social self-concept (*r* = 0.026), or assertiveness (*r* = 0.053), even when age is controlled and especially regarding the frequency of alcohol consumption.

A hierarchical regression analysis has been carried out by taking the level of substance consumption as the dependent variable. It was done in three steps including successively – as independent variables – demographic variables, school well-being variables, and self-concept and self-esteem variables. The resulting equation explains the 37% of variance in the substance consumption level.

Table 4 shows the standardized coefficients and the significance of each independent variable on each of the steps. In the third model, the best sociodemographic predictors are age (*β* = 0.337) and the money available for their expenses (*β* = 0.101). The school well-being variable, which best predicts the consumption level, is educational well-being (*β* = −0.166). In this regression analysis, educational well-being seems to be the main protecting factor against substance consumption. Academic well-being measured as the number of years having been held back (*β* = 0.065), the number of subjects failed (*β* = 0.033), and interpersonal well-being at school (*β* = 0.045) have a minor effect, which seems to favor consumption. Academic self-concept (*β* = −0.105) and self-esteem (*β* = −0.042) seem to have a slight protector effect, whereas assertiveness (*β* = 0.052) and social self-concept (*β* = 0.050) show an equally small effect but is aligned with consumption.

**TABLE 4:** Hierarchical multiple regression for drug use.

	Model 1			Model 2			Model 3		
	*β*	*t*	Sig.	*β*	*t*	Sig.	*β*	*t*	Sig.
(Constant)		–11.765	0.000		–5.229	0.000		–5.037	0.000
Age	0.415	15.548	0.000	0.332	11.951	0.000	0.337	12.219	0.000
Gender	–0.049	–3.232	0.001	–0.018	–1.210	0.226	–0.005	–0.352	0.725
Social level	0.015	0.973	0.331	0.030	1.957	0.050	0.037	2.394	0.017
Money	0.134	7.828	0.000	0.109	6.581	0.000	0.101	6.135	0.000
Bachillerato	0.054	2.623	0.009	0.093	4.472	0.000	0.090	4.367	0.000
FPB	0.090	5.181	0.000	0.094	5.435	0.000	0.097	5.657	0.000
FPM	0.035	1.428	0.153	0.112	4.627	0.000	0.108	4.512	0.000
School well-being				–0.210	–13.850	0.000	–0.166	–10.067	0.000
Academic wb-fails				0.066	3.954	0.000	0.033	1.885	0.059
Academic wb-repet.				0.076	3.620	0.000	0.065	3.116	0.002
Interpersonal wb				0.050	3.334	0.001	0.045	2.906	0.004
Academic self-concept							–0.105	–5.734	0.000
Self-esteem							–0.042	–2.557	0.011
Social self-concept							0.050	2.807	0.005
Assertiveness							0.052	2.912	0.004

	***R***	**a. *R*^2^**	****Δ***R*^2^**	***R***	**a. *R*^2^**	****Δ***R*^2^**	***R***	**a. *R*^2^**	****Δ***R*^2^**

Variance explained	0.553	0.304	0.306	0.600	0.357	0.054	0.611	0.370	0.014

*FPB, Formación Profesional Básica.*

Thirdly, the moderating role of age and gender on the relation between consumption and well-being at school, self-esteem, and self-concept was analyzed. For this purpose, these correlations were calculated separately for each gender and age group (Table 5).

**TABLE 5:** Correlation coefficients of school well-being, self-concept, and self-esteem with drug use moderated by gender and age.

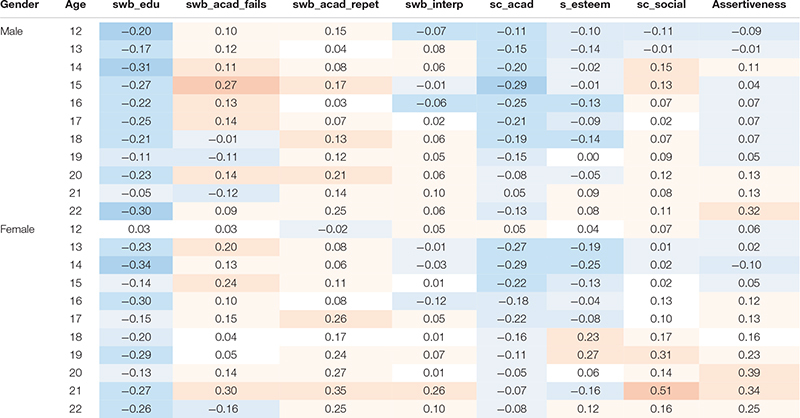

*Blue, negative correlations; Red, positive correlations. The intensity of the color denotes the size of the correlation; the more intense the color, the greater the correlation.*

Academic well-being measured as the number of failed subjects correlates with consumption levels in a different way between the ages: the correlation is small and positive around the age of 13, and it reaches its maximum at the age of 15 and is lower at subsequent ages. This evolution is almost identical in both boys and girls. The evolution of the correlation between consumption and academic well-being measured as the number of years having been held back does not show such clear tendencies, but it seems that it could be increasing with age in the case of girls. The correlation between interpersonal well-being and consumption in boys goes from small positive correlation coefficient values at the age of 13 to slightly negative values at 16, and it regains a slightly positive value at older ages. The evolution of this correlation is similar in girls, but a stronger negative correlation at the age of 16 stands out.

**FIGURE 4:**
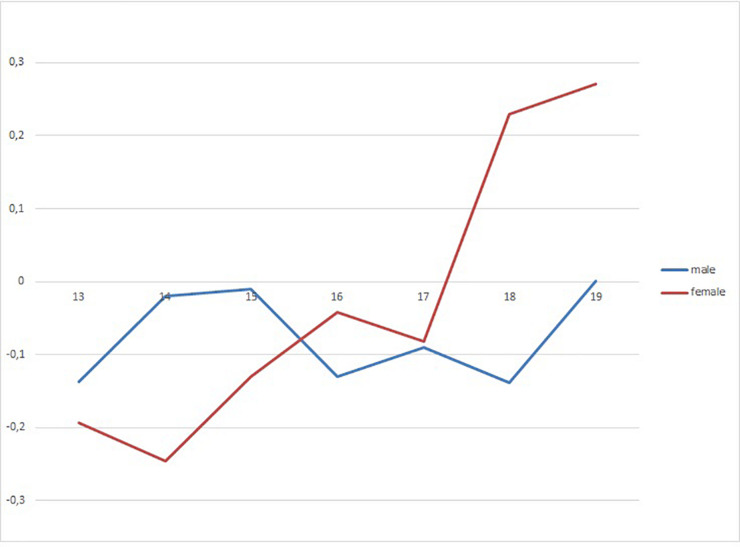
Correlation coefficients of self-esteem with drug use in gender and age groups.

**FIGURE 5:**
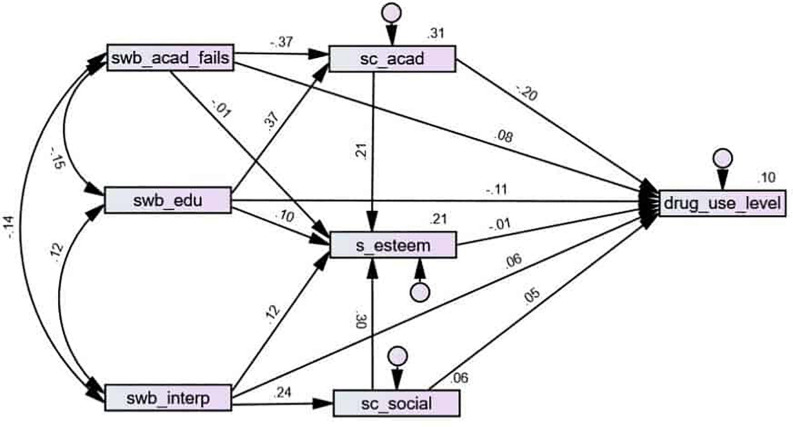
Path analysis for drug use level.

The academic self-concept is correlated with consumption more intensely around the age of 14–15, and it loses strength at older ages. The relation between self-esteem and consumption in boys is slightly negative and stable throughout the years. However, among girls, it goes from negative to positive coefficients throughout the years (Figure 4). Social self-concept shows a positive correlation with consumption at certain times. The correlation coefficient is positive but low for boys aged 14 to 15, whereas for girls, it is higher at the age of 16 and especially from the age of 18 (Figure 5). Assertiveness is correlated with consumption for boys to a lesser extent and in a sustained way, whereas for girls, the association between assertiveness and consumption is higher the older they are.

The mediating role of self-esteem and self-concept in the relation between school well-being and drug use was analyzed using a path analysis (Figure 5) (TLI = 0.907; CFI = 0.982; RMSEA = 0.061). Only cases with no missing answers were used in order to have bootstrapping available for calculating significance levels. Every path was statistically significant, except the one from failing subjects to self-esteem and the one from self-esteem to drug use level.

When calculating the standardized indirect effects of school well-being on drug use level, certain indirect effects correspond to the paths from academic fails (indirect effect, *β* = 0.075; *p* = 0.001) and educational well-being (indirect effect, *β* = −0.075; *p* = 0.001), and much less from interpersonal well-being (indirect effect, *β* = 0.010; *p* = 0.009).

The next step was identifying three specific indirect effects. The indirect effect of fails was significant through academic self-concept (*β* = 0.080; *p* = 0.001), but not through self-esteem (*β* = 0.000; *p* = 0.411). Secondly, the indirect effect of educational well-being was significant through academic self-concept (*β* = −0.079; *p* = 0.001), but not through self-esteem (*β* = 0.001; *p* = 0.348). Finally, the indirect effect of interpersonal well-being through social self-concept was significant but much weaker (*β* = 0.014; *p* = 0.001), and again, it was not significant through self-esteem (*β* = −0.002; *p* = 0.364).

Educational and academic well-being seem to be protecting factors against consumption. This protecting effect is made possible through the reinforcement of academic self-concept. On the contrary, self-esteem does not play a statistically significant mediating role on this protecting effect. Similarly, having better self-esteem does not seem to mediate in the protecting effect of not failing or not being held back years. On the other hand, the indirect effect of interpersonal well-being through social self-concept is small, and nonexistent through self-esteem.

## Discussion

The intention of this article was to analyze the relations between school well-being, self-concept, and self-esteem with consumption in adolescence, as well as the moderating role of age and gender.

This research has proven that educational well-being and academic well-being seem to play a significant protecting role in substance consumption in adolescence. Note that educational well-being has been measured as the perception of good relationships with the teachers and personal involvement in the learning process. The academic well-being variable has been defined objectively by the academic results (number of failed subjects and years having been held back.) These results suggest not only that the greater the educational and academic well-being, the lesser the drug consumption, but also that the consumption of the different substances analyzed would start at a later age. The protecting effect of academic well-being seems clearer in the case of alcohol than in the rest of the substances studied (cannabis, cocaine and stimulant, heroin, and other minority substances). International researches with very relevant samples have reached similar conclusions. For instance, McKay et al. (2012) found these same relationships in Ireland. Low academic self-efficacy was established as the strongest predictor of problematic consumption of alcohol. Maxwell et al. (2017) for their part, have found that the students’ perceptions regarding school environment and their relationship with teachers and with peers significantly reflect on the results obtained in writing and mathematics, even after having controlled the sociodemographic variables. Furthermore, the authors explain that this effect is mediated and reinforced by the students’ psychological identification with the school. Wang and Fredricks (2014) also concluded that students who are committed to school do their homework, participate in class and pay attention, feel more academically competent, are more connected to the institution, and obtain more positive responses, both from the teachers and from their relatives. As a consequence, they also have a lower rate of early school leaving and less behavioral problems, among them, a lower involvement in substance consumption.

Age as a sociodemographic variable has shown a confounding effect at the correlations matrix, which is especially relevant in academic well-being. For example, if the correlation between being held back a year and failing subjects is observed once the effect of age has been controlled, great variations are observed. From this, we may conclude that a fair share of the apparent relationship between bad academic results and consumption may actually be due to the effect of age. However, the protecting effect of educational well-being resists the effect of age, because the correlation between educational well-being and consumption is maintained even when age is controlled. Thus, it continues to be a protecting factor even at the oldest ages where consumptions would be higher.

Academic self-concept and self-esteem have also acted in this research as protective factors in substance consumption. The higher the academic self-concept and self-esteem, the lesser the consumption, although the effect of self-concept is stronger than the effect of self-esteem. Along these lines, Kim et al. (2018) found similar results with consumption and other varied risk conducts. On the contrary, assertiveness seems to act as a risk factor, increasing the probability of consumption.

Along with the protecting role, a mediation effect of academic self-concept and social self-concept between school well-being and consumption has also been found. Thus, the effect of academic well-being and educational well-being on consumption would have an impact through academic self-concept, whereas the effect of social well-being on consumption would be less mediated by social self-concept.

Some of these relationships are moderated by gender and age. Thus, academic self-concept protects more around the age of 14/15, and from that age, its protecting power decreases, in both boys and girls. In the case of self-esteem, it protects a little, but more constantly in the case of boys. In the case of girls, however, it acts more irregularly; between the ages of 13 and 14, it plays a protecting role, whereas between the ages of 18 and 19, it is a risk factor. The assertiveness variable has a similar behavior. It assumes a risk role, which is stable in time, although it is also lower in boys; but again in girls, at the age of 14 it protects and from the age of 15, it becomes a growing risk factor. There are several studies on this last line of work: social competence, social skills, and assertiveness. On an international level, McKay et al. (2012) found that high social self-efficacy was a predictor of problematic alcohol consumption. On a state level, several studies (Cava et al., 2008; Jiménez-Rodrigo, 2008; Jiménez, 2011; Jiménez et al., 2008) refer to the risk effect of social self-esteem. Some authors explain this, affirming that high social self-esteem offers more opportunities to experiment and socialize with peers and, therefore, consume (Sánchez-Sosa et al., 2014). And this consumption in adolescents is associated with identification processes, embracing values and group attitudes. Regarding assertiveness as a risk factor, it has been confirmed by other researches such as the one developed by Hernández-Serrano et al. (2016). On the contrary, Fuentes et al. (2011) have found that these positive relations disappear when the risk and gender effects are controlled. In any case, the fact that adolescents feel socially able and are perceived or express themselves behaviorally as such, and this being related to substance consumption, constitute a challenge for socio-educational and psycho-educational intervention.

Although the educational itinerary is not the main objective of this study, the results from the first objective indicate that the average level of consumption per stage is strongly conditioned by age. The average consumption without controlling age is much higher starting from ESO, but the resulting average after controlling age is not. In fact, the school context with the highest consumption once age is controlled is FPB (basic vocational training), which sets out the challenge of a socio-educational intervention in this specific context and the education needs of this educational itinerary.

Lastly, the analysis of age in relation to consumption makes it possible to affirm that this risk conduct may be just another experimental conduct. Consumptions decrease after peaking. However, it is necessary to wait until the start of early manhood and womanhood (twenties) for these consumptions to decrease. The most minor or marginal substances will mark a “red line,” which is a warning call or a risk factor. The prevalence indicators are higher the older in age, with the highest consumptions found at the age of 19 and 20, and decreasing in the following years. The ages of greater irruption of each substance are different: earlier for alcohol consumption, followed by cannabis, stimulant substances, and minority substances (heroin, LSD, and other similar substances). The idea that age might play an especially relevant role in consumption and in adolescence, as a risk conduct and as experimental consumption (González de Audikana, 2016) had been indicated at the start of the article. Sánchez-Sosa et al. (2014) argued that drug consumption in adolescence is another way of experimenting, as are romantic and sexual relationships, social relations, and power relations. In fact, they put to the test the environment and organization of the family and the school, both of which have the power of favoring or avoiding further consumption escalation. Tena-Suck et al. (2018) also affirm that adolescents are at a higher risk of consuming substances than adults. Nevertheless, the percentage is very small, because no more than 30% consume, and a very small percentage of those do so with harmful patterns or with dependency indicators. Along the same lines, INJUVE (2017) presented in its report a “photograph” of adolescence and early manhood and womanhood. Most adolescents are not involved in risk conducts, nor in violent acts of any kind.

To end the discussion, we must refer to the sociodemographic variable of gender, although we have been inevitably referring to it throughout this section. In this research, girls have a greater educational and academic well-being, as well as a better academic self-concept than boys. However, they show slightly lower self-esteem, social self-concept, and assertiveness levels than do boys. Regarding consumptions, girls have the highest percentages of abstinents and alcohol consumers (this latter group with numbers close to the boys’), but they also consume less minority drugs and other drugs. When observing the presence of consumptions in the last month, boys exceed girls in all substances. Hernández-Serrano et al. (2016) also found similar relations, both in age and in relation to gender. The relation with age is positive and direct, and girls consume more alcohol than boys. Riquelme et al. (2018) confirmed the action of both sociodemographic variables: the effect of age and gender. Older adolescents presented a greater substance consumption of cannabis and synthetic drugs, but this difference was not found in adolescent girls, which aligns perfectly with the results and conclusions found in this research.

### Limitations

Although this research includes a representative, stratified, and random sample, it is a transversal and correlational study. Although the students of different ages are adequately represented, it is not a longitudinal design, which would allow to see the evolution of the same people during a period of time and to get closer to establishing causality relations. Consequently, the interpretations of the tendencies of the variables at the different ages may only propose temporary sequences and causal relationships in a tentative way.

On the other hand, the sample is composed of students from secondary education and vocational training between the ages of 12 and 22; therefore, its representativeness is limited from the age of 16, considering that it does not include the people who might have abandoned the educational system from this age on or those who are studying at university from the age of 18.

## Conclusion

Three main conclusions may be drawn from this research. Firstly, that educational well-being, academic well-being, academic self-concept, and self-esteem seem to play a role as protective factors in adolescence. In order to confirm this, longitudinal research designs are necessary.

Secondly, that academic self-concept plays a mediating role between school well-being and consumption, meaning that the effect of academic and educational well-being takes place through the reinforcement of academic self-concept. By contrast, assertiveness influences but tends to do so negatively, being associated with a greater risk of consumption. Some of these relationships are moderated by the variables of gender and age.

Thirdly, age and gender are very relevant sociodemographic variables to consider in order to understand this phenomenon. Age has shown its moderating effect, especially relevant in the effect of academic well-being. It has also proved to be important in order to understand its experiential or experimental and transitory nature. Differences in consumptions based on gender have also been found.

One of the main implications that may derive from this research is that these variables, but mainly educational well-being, are in the hands of educational agents, which means that relationships, the participation of the students, the environment, the rules of coexistence, the size of the group, the ways of solving conflicts, the tutoring, and the school organization are all variables that may be influenced and modified.

## Data Availability Statement

The datasets analyzed in this article are not publicly available due to commitments to confidentiality and anonymity of responses. Requests to access the datasets should be directed to josu.solabarrieta@deusto.es.

## Ethics Statement

The studies involving human participants were reviewed and approved by the Ethics Committee of the University of Deusto (Ref.: ETK-31/16-17). Aspects such as the specific ways in which research participants will be recruited, the application of informed consent procedures, and the implementation of personal data management and interaction with vulnerable people are all suitably explained according to the main ethical standards. Detailed information shall be provided to the participants by means of an information and the informed consent sheet including descriptions/specifications of the following: purpose of the research; duration of the research activities; adopted procedures; voluntary participation; possible risks, discomfort, or disadvantages; benefits to the subject or others; data protection and confidentiality and privacy policies; where to get more information; and what happens to data, samples, and results at the end of the research.

## Author Contributions

All authors listed have made a substantial, direct and intellectual contribution to the work, and approved it for publication. RS and MR-N carried out the literature review. RS prepared the introductory and discussion sections. MR-N supported the process and reviewed all references. JS conducted the data analysis and results section. All authors were involved in the entire process, reviewing and contributing to all parts of the written manuscript.

## Conflict of Interest

The authors declare that the research was conducted in the absence of any commercial or financial relationships that could be construed as a potential conflict of interest.

## Footnotes

1 They define self-efficacy according to Bandura’s definition (1997) as the belief in one’s own ability to exercise control over challenging demands and functioning.

## References

[B1] Aiken L. S. West S. G. (1991). *Multiple Regression: Testing and Interpreting Interactions.* Thousand Oaks, CA: Sage Publications, Inc.

[B2] Álvarez-García D. González-Castro P. Núñez J. C. Rodríguez C. Cerezo R. (2019). Impact of family and friends on antisocial adolescent behavior: the mediating role of impulsivity and empathy. *Front. Psychol.* 10:2071. 10.3389/fpsyg.2019.02071 31551894 PMC6746981

[B3] Bandura A. (1997). *Self efficacy: The Exercise of Control.* New York, NY: Freeman.

[B4] Batllori A. (2016). *El Consumo de Drogas Entre Adolescentes: Prevención en la Escuela y en la Familia.* Madrid: Narcea Ediciones.

[B5] Benner A. D. Wang Y. (2015). Adolescent substance use: the role of demographic marginalization and socioemotional distress. *Dev. Psychol.* 51 1086–1097. 10.1037/dev0000026 26075631 PMC4516632

[B6] Blanco A. Díaz D. (2005). Social well-being: theoretical structure and measurement. *Psicothema* 17 582–589.

[B7] Cash A. Bradshaw C. Philip J. (2014). Observations of student behavior in nonclassroom settings: a multilevel examination of location. Density, and School Context. *J. Early Adolesc.* 35 597–627. 10.1177/0272431614562835

[B8] Cava M. Murgui S. Musitu G. (2008). Diferencias en factores de protección del consumo de sustancias en la adolescencia temprana y media. *Psicothema* 20 389–395. 18674432

[B9] CEE (2018). *Informe 2018 Sobre el Estado del Sistema Educativo. Curso 2016-2017.* Madrid: Ministerio de Educación y Formación Profesional.

[B10] Chen Y. Rendina-Gobioff G. Dedrick R. F. (2007). Detecting effects of positively and negatively worded items on a self-concept scale for third and sixth grade elementary students. 52nd annual meeting of the florida educational research association. *Tampa U. S.* 1:2007.

[B11] Christens B. D. Peterson N. A. (2012). The role of empowerment in youth development: a study of sociopolitical control as mediator of ecological systems’ influence on developmental outcomes. *Youth Adolesc.* 41 623–635. 10.1007/s10964-011-9724-9 22038436

[B12] Collison D. Banbury S. Lusher J. (2016). Relationships between age, sex, self-esteem and attitudes towards alcohol use amongst university students. *J. Alcohol Drug Educ.* 60 16–34.

[B13] Connolly J. Josephson W. Schnoll J. Simkins-Strong E. Pepler D. MacPherson A. (2015). Evaluation of a youth-led program for preventing bullying, sexual harassment, and dating aggression in middle schools. *J. Early Adolesc.* 35 403–434. 10.1177/0272431614535090

[B14] De Boer A. Peeters M. Koning I. (2016). An experimental study of risk taking behavior among adolescents: a closer look at peer and sex influences. *J. Early Adolesc.* 37 1125–1141. 10.1177/0272431616648453

[B15] DuBois D. L. Silverthorn N. (2004). Bias in self-perceptions and internalizing and externalizing problems in adjustment during early adolescence: a prospective investigation. *J. Clin. Child Adolesc. Psychol.* 33 373–381. 10.1207/s15374424jccp3302_19 15136202

[B16] EMCDDA (2015). *Section 2. Analysis of Risk and Protective Factors.* Lisbon: EMCDDA 10.1207/s15374424jccp3302_19

[B17] ESPAD Group (2016). *ESPAD Report 2015: Results From the European School Survey Project on Alcohol and Other Drugs.* Luxembourg: Publications Office of the European Union.

[B18] Fuentes M. C. García J. F. Gracia E. Lila M. (2011). Autoconcepto y ajuste psicosocial en la adolescencia. *Psicothema* 23 7–12. 21266135

[B19] Fuller-Thomson E. Sheridan M. P. Sorichetti C. Mehta R. (2013). Underage binge drinking adolescents: sociodemographic pro?le and utilization of family doctors. *ISRN Fam. Med.* 2013:728730. 10.5402/2013/728730 24959572 PMC4041245

[B20] García del Castillo J. A. Lloret D. Espada J. P. (2004). Prevalencia del consumo de tobacco en población universitaria. *Salud Drogas* 4 19–28.

[B21] Golpe S. Isorna M. Barreiro C. Braña R. Rial A. (2017). Consumo intensivo de alcohol en adolescentes: prevalencia, conductas de riesgo y variables asociadas. *Adicciones* 29 256–267. 28170056 10.20882/adicciones.932

[B22] González de Audikana M. (2008). “Siete claves para entender lo que ha pasado en estos 25 años con el consumo de drogas entre nuestros escolares,” in *Las drogas entre los Escolares de Euskadi, Veinticinco años Después*, eds Elzo J. Laespada M. T. Drogas y Escuela VII (Bilbo: Universidad de Deusto), 2008.

[B23] González de Audikana M. (2016). *Adolescencia, fracaso escolar y consumo de drogas (Tesis Doctoral).* Bilbao: Universidad de Deusto.

[B24] González de Audikana M. (2017). “Un ejemplo de reciclaje de dos investigaciones para su aprovechamiento en la aplicación de intervenciones preventivas,” in *Repensando la Prevención*, ed. González de Audikana M. (Bilbao: Universidad de Deusto).

[B25] González de Audikana M. Laespada M. (2014). “La prevención selectiva en el ámbito educativo y las buenas prácticas,” in *Prevención de las Drogodependencias y otras Conductas Adictivas*, eds Isorna M. Saavedra D. (Madrid: Pirámide), 319–346.

[B26] Good M. Willoughby T. (2014). Institutional and personal spirituality/religiosity and psychosocial adjustment in adolescence: concurrent and longitudinal associations. *J. Youth Adolesc.* 43 757–774. 10.1007/s10964-013-9989-2 23955323

[B27] Greenberger E. Chen C. Dmitrieva J. Farruggia S. P. (2003). Item-wording and the dimensionality of the rosenberg self-esteem scale: do they matter? *Personal. Individ. Differ.* 35 1241–1254. 10.1016/S0191-8869(02)00331-8

[B28] Harter S. (2012). *Self-Perception Profile for Adolescents: Manual and Questionnaires.* Denver, CO: University of Denver.

[B29] Hernández-Serrano O. Espada J. Guillén-Riquelme A. (2016). Relación entre conducta prosocial, resolución de problemas y consumo de drogas en adolescentes. *Anal. Psicol.* 32 609–616. 10.6018/analesps.32.2.204941

[B30] Huang C. Dong N. (2012). Factor structures of the rosenberg self-esteem scale: a meta-analysis of pattern matrices. *Eur. J. Psychol. Assess.* 28 132–138. 10.1027/1015-5759/a000101

[B31] INJUVE (2017). *Informe Juventud en España 2016.* Madrid: Editor.

[B32] Instituto Deusto Drogodependencias (2019). *Drogas y Escuela IX.* Bilbao: Publicaciones Universidad de Deusto.

[B33] ISEI-IVEI (2017). *Irakas Sistema Ebaluatu eta Ikertzeko Erakundea – Instituto Vasco de Evaluación e Investigación Educativa. PISA 2015 Euskadi. Informe de Resultados.* Bilbao: ISEI-IVEI.

[B34] Jiménez T. I. (2011). Risk and protective self-esteem: a mediational role between family environment and substance use in adolescents/autoestima de riesgo y protección: una mediación entre el clima familiar y el consumo de sustancias en adolescentes. *Psychosoc. Interv.* 20 53–61. 10.5093/in2011v20n1a5

[B35] Jiménez T. I. Musitu G. Murgui S. (2008). Funcionamiento familiar y consumo de sustancias en adolescentes: el rol mediador de la autoestima. *Int. J. Clin. Health Psychol.* 8 139–151.

[B36] Jiménez-Rodrigo M. L. (2008). Una profecía que se cumple a sí misma: tras los mitos del consumo femenino adolescente de cigarrillos. *Liberaddictus* 101 11–16.

[B37] Keyes C. L. M. (1998). Social well-being. *Soc. Psychol. Q.* 61 121–140.

[B38] Keyes C. L. M. Shmotkin D. Ryff C. D. (2002). Optimizing well-being: the empirical encounter of two traditions. *J. Pers. Soc. Psychol.* 82 1007–1022. 10.1037/0022-3514.82.6.1007 12051575

[B39] Khan M. R. Cleland C. M. Scheidell J. D. Berger A. T. (2014). Gender and racial/ethnic differences in patterns of adolescent alcohol use and associations with adolescent and adult illicit drug use. *Am. J. Drug Alcohol Abuse* 40 213–224. 10.3109/00952990.2014.892950 24766088

[B40] Kim D. H. Bassett S. M. Takahashi L. Voisin D. R. (2018). What does self-esteem have to do with behavioral health among low-income youth in Chicago? *J. Youth Studi.* 21 999–1010. 10.1080/13676261.2018.1441982

[B41] Konu A. Alanen E. Lintonen T. Rimpelä M. (2002). Factor structure of the school well-being model. *Health Educ. Res. Theory Pract.* 17 732–742. 10.1093/her/17.6.732 12507348

[B42] Konu A. Joronen K. Lintonen T. (2015). Seasonality in school well-being: the case of finland. *Child Res.* 8 265–277. 10.1007/s12187-014-9243-9

[B43] Lázaro-Visa S. Palomera R. Briones E. Fernández-Fuertes A. A. Fernández-Rouco N. (2019). Bullied adolescent’s life satisfaction: personal competencies and school climate as protective factors. *Front. Psychol.* 10:1691. 10.3389/fpsyg.2019.01691 31379695 PMC6657649

[B44] Li Y. Lerner R. (2011). Trajectories of school engagement during adolescence: implications for grades, depression, delinquency, and substance use. *Dev. Psychol.* 47 233–247. 10.1037/a0021307 21244162

[B45] Little T. D. Cunningham W. A. Shahar G. Widaman K. F. (2002). To parcel or not to parcel: exploring the question, weighing the merits. *Struct. Equ. Modeling* 9 151–173. 10.1207/S15328007SEM0902_1

[B46] Malonda E. Llorca A. Mesurado B. Samper P. Mestre M. V. (2019). Parents or peers? Predictors of prosocial behavior and aggression: a longitudinal study. *Front. Psychol.* 10:2379. 10.3389/fpsyg.2019.02379 31695656 PMC6817951

[B47] Maxwell S. Reynolds K. J. Lee E. Subasic E. Bromhead D. (2017). The impact of school climate and school identification on academic achievement: multilevel modeling with student and teacher data. *Front. Psychol.* 8:2069. 10.3389/fpsyg.2017.02069 29259564 PMC5723344

[B48] McKay M. T. Sumnall H. Goudie A. J. Field M. Cole J. C. (2011). What differentiates adolescent problematic drinkers from their peers? Results from a cross-sectional study in Northern Irish school children. *Drugs* 18 187–199. 10.3109/09687637.2010.502160

[B49] McKay M. T. Sumnall H. R. Cole J. C. Percy A. (2012). Self-esteem and self-efficacy: associations with alcohol consumption in a sample of adolescents in Northern Ireland. *Drugs* 19 72–80. 10.3109/09687637.2011.579585

[B50] MEFP (2019). *Ministerio de Educación y Formación Profesional. Sistema estatal de indicadores de la Educación 2019.* Madrid: Ministerio de Educación y Formación Profesional.

[B51] Moral J. C. Sánchez J. C. Villarreal M. E. (2010). Desarrollo de una escala multidimensional breve de ajuste escolar. *Rev. Electr. Metodol. Apl.* 15 1–11.

[B52] Motos P. Cortés M. Giménez J. Cadaveira F. (2015). Predictores del consumo semanal de alcohol y sus consecuencias asociadas en universitarios consumidores intensivos de alcohol. *Adicciones* 27 119–131.26132301

[B53] Observatorio Español de las Drogas y las Adicciones (2019). *Encuesta sobre Uso de Drogas en Enseñanzas Secundarias en España (ESTUDES) 1994-2018.* Madrid: Ministerio de Sanidad, Consumo y Bienestar Social.

[B54] Páramo M. A. Leo M. K. Cortés M. J. Morresi G. M. (2015). Influencia del bienestar psicológico en la vulnerabilidad a conductas adictivas en adolescentes escolarizados de 15 a 18 años. *Rev. Argent. Clín. Psicol.* 24 167–178.

[B55] Peñafiel E. (2009). Factores de riesgo y protección en el consumo de sustancias en adolescentes. *Pulso Revi. Educ.* 32 147–173.

[B56] Peralta R. L. Steele J. L. Nofziger S. Rickles M. (2010). The impact of gender on binge drinking behavior among US college students attending a Midwestern university: an analysis of two gender measures. *Fem. Criminol.* 5 355–379. 10.1177/1557085110386363

[B57] Rathus S. (1973). Thirty-item schedule for assessing assertive behavior. *Behav. Ther.* 4 398–406. 10.1016/s0005-7894(73)80120-0

[B58] Rial A. Burkhart G. Isorna M. Barreiro C. Varela J. Golpe S. (2019). Consumo de cannabis entre adolescentes: patrón de riesgo, implicaciones y posibles variables explicativas. *Adicciones* 31 64–77. 30059583 10.20882/adicciones.1212

[B59] Riquelme M. García O. Serra E. (2018). Desajuste psicosocial en la adolescencia: socialización parental, autoestima y uso de sustancias. *Anal. Psicol.* 34 536–544. 10.6018/analesps.34.3.315201

[B60] Rosenberg M. (1965). Rosenberg self-esteem scale (RSE): acceptance and commitment therapy. *Measur. Pack* 61:52.

[B61] Ryff C. (1989). Happiness is everything, or is it? Explorations on the meaning of psychological well-being. *J. Pers. Soc. Psychol.* 57 1069–1081. 10.1037/0022-3514.57.6.1069

[B62] Ryff C. Keyes C. L. M. (1995). The structure of psychological well-being revisited. *J. Personal. Soc. Psychol.* 69 719–727. 10.1037/0022-3514.69.4.719 7473027

[B63] Sánchez-Sosa J. Villarreal-González M. Ávila M. Vera A. Musitu G. (2014). Contextos de socialización y consumo de drogas ilegales en adolescentes escolarizados. *Psychosoc. Interv.* 23 69–78. 10.5093/in2014a7

[B64] Sarkova M. Bacikova-Sleskova M. Geckova A. M. Katreniakova Z. van den Heuvel W. van Dijk J. P. (2014). Adolescents’ psychological well-being and self-esteem in the context of relationships at school. *Educ. Res.* 56 367–378. 10.1080/00131881.2014.965556

[B65] Skogen J. C. Sivertsen B. Hysing M. Heradstveit O. Bøe T. (2019). Economic circumstances in childhood and subsequent substance use in adolescence – a latent class analysis: the youth@hordaland study. *Front. Psychol.* 10:1115. 10.3389/fpsyg.2019.01115 31139128 PMC6527884

[B66] Tena-Suck A. Castro-Martínez G. Marín-Navarrete R. Gómez-Romero P. (2018). Consumo de sustancias en adolescentes: consideraciones para la práctica médica. *Med. Int. Méx.* 34 264–277. 10.24245/mim.v34i2.1595

[B67] Wang M. Eccles J. S. (2012). Social support matters: longitudinal effects of social support on three dimensions of school engagement from middle to high school. *Child Dev.* 83 877–895. 10.1111/j.1467-8624.2012.01745.x 22506836

[B68] Wang M. Fredricks J. (2014). The reciprocal links between school engagement youth problem behaviors, and school dropout during adolescence. *Child Dev.* 85 722–737. 10.1111/cdev.12138 23895361 PMC3815520

[B69] Wheeler S. (2010). Effects of self-esteem and academic performance on adolescent decision-making: an examination of early sexual intercourse and illegal substance use. *J. Adolesc. Health* 47 582–590. 10.1016/j.jadohealth.2010.04.009 21094435

